# Gene mutations as a non-invasive measure of adult cochlear implant performance: Variable outcomes in patients with select TMPRSS3 mutations

**DOI:** 10.1371/journal.pone.0291600

**Published:** 2023-09-15

**Authors:** Justin Cottrell, Peter Dixon, Xingshan Cao, Alex Kiss, Kari Smilsky, Kassandra Kaminskas, Amy Ng, David Shipp, Andrew Dimitrijevic, Joseph Chen, Vincent Lin, Lianna Kyriakopoulou, Trung Le

**Affiliations:** 1 Department of Otolaryngology–Head and Neck Surgery, University of Toronto, Toronto, Ontario, Canada; 2 Department of Research Design and Biostatistics, Sunnybrook Health Sciences Centre, Toronto, Ontario, Canada; 3 Department of Laboratory Medicine & Pathology–Clinical Chemistry, Hospital for Sick Children, Toronto, Ontario, Canada; Shaheed Rajaei Hospital: Rajaie Cardiovascular Medical and Research Center, ISLAMIC REPUBLIC OF IRAN

## Abstract

**Background:**

The cochlear implant (CI) has proven to be a successful treatment for patients with severe-to-profound sensorineural hearing loss, however outcome variance exists. We sought to evaluate particular mutations discovered in previously established sensory and neural partition genes and compare post-operative CI outcomes.

**Materials and methods:**

Utilizing a prospective cohort study design, blood samples collected from adult patients with non-syndromic hearing loss undergoing CI were tested for 54 genes of interest with high-throughput sequencing. Patients were categorized as having a pathogenic variant in the sensory partition, pathogenic variant in the neural partition, pathogenic variant in both sensory and neural partition, or with no variant identified. Speech perception performance was assessed pre- and 12 months post-operatively. Performance measures were compared to genetic mutation and variant status utilizing a Wilcoxon rank sum test, with P<0.05 considered statistically significant.

**Results:**

Thirty-six cochlear implant patients underwent genetic testing and speech understanding measurements. Of the 54 genes that were interrogated, three patients (8.3%) demonstrated a pathogenic mutation in the neural partition (within TMPRSS3 genes), one patient (2.8%) demonstrated a pathogenic mutation in the sensory partition (within the POU4F3 genes). In addition, 3 patients (8.3%) had an isolated neural partition variance of unknown significance (VUS), 5 patients (13.9%) had an isolated sensory partition VUS, 1 patient (2.8%) had a variant in both neural and sensory partition, and 23 patients (63.9%) had no mutation or variant identified. There was no statistically significant difference in speech perception scores between patients with sensory or neural partition pathogenic mutations or VUS. Variable performance was found within patients with TMPRSS3 gene mutations.

**Conclusion:**

The impact of genetic mutations on post-operative outcomes in CI patients was heterogenous. Future research and dissemination of mutations and subsequent CI performance is warranted to elucidate exact mutations within target genes providing the best non-invasive prognostic capability.

## Introduction

Hearing loss is the most common sensory disorder with approximately 360 million people, 5% of the world’s population, having a disabling hearing impairment as per estimates by the World Health Organization (WHO) [1]. The economic impact is an estimated $750 billion dollars globally for unaddressed hearing loss, with additional impacts for patients including possible social isolation, communication difficulty, cognitive decline and dementia [2].

Recent human genetic research attributes a genetic cause to 60–80% of deafness in developed countries [3]. Both syndromic and non-syndromic hearing loss cases are generally monogenic disorders, but with large genetic heterogeneity. To date, over 124 genes have been identified as causing nonsyndromic hearing loss [4]. Estimates suggest that >250 genes may be involved for syndromic and nonsyndromic inheritance patterns respectively, with many genes remaining to be identified [5].

The peripheral auditory system can be divided into the neural partition (spiral ganglion) and sensory partition (organ of Corti and synapse). A cochlear implant (CI) bypasses the sensory partition and stimulates the neural partition directly, therefore, if the health of the neural partition is compromised, this may result in worse CI performance [6]. CI recipients for severe-to-profound sensorineural hearing loss demonstrate a wide range in speech performance, possibly driven by the degree of neural degeneration [6, 7]. Previous literature using Combined Annotation Dependent Depletion (CADD) score analysis to identify pathogenic variants has suggested patients with pathogenic variants in the neural partition of hearing have worse post-operative outcomes [6]. We sought to better understand the role of specific genetic mutations found in non-syndromic hearing loss on adult cochlear implant performance, and better elucidate the relationship between genetic variants in the sensory and neural partition utilizing the American College of Medical Genetics and Genomics (ACMG) criteria for pathogenic variant classification [8].

## Materials and methods

The study protocol met Research Ethic Board Approval (REB #3871–001) on February 14, 2018, and was renewed throughout the study period, by the Research Ethics Board of Sunnybrook Health Sciences Centre. Cochlear implant candidates over the age of 18 years of age with bilateral post-lingual, non-syndromic sensorineural hearing loss meeting cochlear implant criteria were contacted to discuss consenting for study participation at Sunnybrook Health Sciences Centre. All eligible patients meeting inclusion criteria were approached for participation. Patients providing written consent had blood samples collected for genetic analysis, underwent cochlear implantation and subsequent post-operative performance evaluation. The results of the genetic analysis were provided to patients through pre-existing genetic counselling resources available through the cochlear implant program. Participant recruitment was initiated March 2018 and was completed by January 2021. Study participant blood samples and medical record data was collected between March 2018 and May 2023. Authors had access to identifiable patient information at time of data collection for each patient, and then data was anonymized without identifiers, for analysis.

Speech audiometric evaluation included three standard measures of speech understanding: the AzBio sentence, HINT (Hearing in Noise Test), and CNC test (Consonant-Vowel Nucleus-Consonant) [9–11]. HINT and AzBio were performed both in quiet and in noise (+5 dB signal to noise ratio) environments. Post-operative audiologic assessments occurred at 3-, 6- and 12-months following implantation, and then annually after the first year. One-year audiologic results were used for comparison of outcomes, however the most recent test result was used when one-year result was not available. An average composite z-score of all audiometric results was calculated as an additional metric to complete statistical analysis.

Blood samples were tested using high-throughput Next Generation Sequencing (NGS) to identify variants in 54 genes that have been implicated in sensory or neural partition pathology (Table 1) [6]. Final genes selected for study inclusion were drawn from prior research by Shearer *et al*., in addition to genes included in OtoSCOPE at the time of initial patient recruitment (2018), taking into account the study site’s genetic sequencing capabilities [6, 12]. Additional genes discovered over the course of the study were not added to the panel, to improve standardization between patients. Next Generation sequencing was performed using Agilent SureSelect capture followed by paired-end sequencing using the Illumina sequencing platform. Variant calls were generated using Genomic Analysis Tool Kit (GATK) after read alignment with the Burrows-Wheeler Aligner (BWA) to genome build GRCh37/UCSC hg19. Intronic regions are covered only within +/-10bp of the splice junction and therefore variants within regulatory domains or deep intronic regions were not assessed.

**Table 1:** List of interrogated genes with partition classification.

Interrogated Sensory Partition Genes	Interrogated Neural Partition Genes
*ACTGY1*	*AIFM1*
*ADCY1*	*CABP2*
*CEACAM16*	*CHD7*
*CLDNI4*	*CLRN1*
*CLIC5*	*DFNB59*
*EPS8*	*DIAPH3*
*GPSM2*	*EDN3*
*ILDR1*	*ESRRB*
*LOXHD1*	*EYA1*
*MYH9*	*GIPC3*
*MYHI4*	*KARS*
*MYO6*	*MIR96*
*MYO15A*	*MSRB3*
*OTOA*	*MTRNR1*
*OTOGL*	*OPA1*
*POU4F3*	*OTOF*
*PTPRQ*	*P2RX2*
*RDX*	*PAX3*
*SERPINB6*	*PNPT1*
*SIX1*	*PRPS1*
*SMPX*	*SLC17A8*
*STRC*	*SOX10*
*TMIE*	*SYNE4*
*TPRN*	*TBC1D24*
*TRIOBP*	*TMC1*
*USH1C*	*TMPRSS3*
	*TSPEAR*
	*WSF1*

Variants were classified as benign, pathogenic or variants of unknown significance (VUS) using the ACMG guidelines [8]. Assessment of the clinical significance of a variant, was performed using control population data (dbSNP, 1000 Genomes Project, Exome Variant Server (EVS), and the Exome Aggregation Consortium (ExAC)), functional data, computational data (PolyPhen2, SIFT, Align GVGD and MutationTaster) to provide predictions about the effect of missense changes that result in the translation of a different amino acid at that position, segregation data and databases describing previously reported variants such as the Human Gene Mutation Database (HGMD) and ClinVar, as well as relevant published scientific literature. We did not include autosomal recessive mutations for carriers only (i.e. if a patient had a single copy). Following variant identification and pathogenicity determination, patients were categorized as having a pathogenic variant in the sensory partition, pathogenic variant in the neural partition, pathogenic variant in both sensory and neural partition, or with no variant identified. Furthermore, patients scoring one standard deviation below the average had their variants interrogated to determine whether a variant of unknown significance could be re-classified as a new pathogenic variant, and to better understand particular genetic mutations and their preponderance for poor post-operative performance.

The audiometric findings were compared to genetic mutation and variant status. Wilcoxon rank sum test was completed using SAS OnDemand for Academics with P value < 0.05 considered to be statistically significant. The primary endpoint was difference in average post-operative combined audiometric z-score. Secondary endpoints included difference in average post-operative CNC, AzBio (quiet and noise), and HINT (quiet and noise) scores respectively. The primary patient comparisons were patients demonstrating a known pathogenic variant in the sensory and neural partition. Secondary patient comparisons included patients with known pathogenic variants in the neural partition against all other patients (with and without identified mutations); comparing patients with known pathogenic mutations or variants of unknown significance (VUS) in the neural partition to those with pathogenic mutations or VUS in the sensory partition; and comparing patients with pathogenic mutations or VUS in both neural and sensory partition compared to patients with a single mutation.

## Results

Thirty-six patients met study criteria, with an average age of 56.4 years (Stdev: 12.5) at time of implantation (Table 2). Of the 54 genes that were interrogated, three patients (8.3%) demonstrated a pathogenic mutation in the neural partition (within TMPRSS3 genes) and one patient (2.8%) demonstrated a pathogenic mutation in the sensory partition (within the POU4F3 gene). In addition, 3 patients (8.3%) had an isolated neural partition VUS, 5 patients (13.9%) had an isolated sensory partition VUS, 1 patient (2.8%) had a variant in both neural and sensory partition, and 23 patients (63.9%) had no diagnostic variant(s) identified (Table 3).

**Table 2:** Demographic characteristics of the studied patients.

	n (%)
**Patients Included**	36 (100%)
**Sex**	
Male	17 (47.2%)
Female	19 (52.8%)
**Age**	**Mean (stdev) **
Age at Implant	56.4 (12.5)

**Table 3:** Mean post-operative performance in relation to patient genetic profile.

	Patients Identified (n)	Azbio Quiet		AzBio Noise		HINT Quiet		HINT Noise		CNC		Combined Z Score	
Patient Cohort		n	Mean Score (Std Dev)	n	Mean Score (Std Dev)	n	Mean Score (Std Dev)	n	Mean Score (Std Dev)	n	Mean Score (Std Dev)	n	Mean Score (Std Dev)
All Patients	36	33	85 (16.6)	31	57 (28.0)	27	91 (12.7)	25	71 (21.3)	36	61 (19.2)	36	-0.04 (0.91)
Sensory Partition Pathogenic Mutation	1	1	94 (-)	1	92 (-)	1	89 (-)	1	66 (-)	1	60 (-)	1	0 (-)
Neural Partition Pathogenic Mutation	3	3	84.3 (13.1)	3	63.7 (35.1)	2	91 (1.4)	2	52 (56.6)	3	68.7 (16.8)	3	0.2 (1.1)
Isolated Sensory Partition Variance of Unknown Significance	5	5	95.2 (4.4)	5	68.6 (23.2)	5	97.4 (3)	4	81.3 (15.6)	5	70.4 (15.1)	5	0.5 (0.4)
Isolated Neural Partition Variance of Unknown Significance	3	3	82.3 (11.2)	3	62 (19.3)	3	92 (5.6)	3	79.7 (11.2)	3	64 (19.3)	3	0.1 (0.4)
Neural and Sensory Partition Variance of Unknown Significance	1	1	93 (-)	1	50 (-)	1	98 (-)	1	61 (-)	1	64 (-)	1	0.1 (-)
No Mutation	23	20	82.5 (19.7)	18	50.2 (29.8)	15	88.7 (16.3)	14	69.4 (19.9)	23	57 (20.9)	23	-0.2 (1.0)

Twenty-seven patients (75.0%) had audiometric data available for the HINT in quiet test, 25 patients (69.4%) for HINT in noise, 33 patients (91.7%) for AZBIO in quiet, 31 patients (86.1%) for AZBIO in noise, and 36 patients (100%) for CNC scores. Twenty-two patients (61.1%) had available data across all audiometric tests (Table 3)

There was no statistical difference when comparing outcomes between patients with pathogenic mutations in the neural or sensory partitions identified (P value > 0.05). There was also no statistical difference in audiological outcomes in the other patient group comparisons. After retrospectively interrogating variants in the patients demonstrating poor audiometric outcomes (Z <1), no VUS met criteria to be reclassified as a pathogenic variant. A list of pathogenic and VUS mutations, with their corresponding speech performance can be found in Tables 4 and 5.

**Table 4:** Post-operative performance of patients with identified pathogenic mutations.

		Pathogenic Mutations		Post-Operative Performance					
Partition	Patient #	Gene	Mutation	AzBio Quiet	AzBio Noise	HINT Quiet	HINT Noise	CNC	Combined Z Score
Sensory	1	POU4F3	c.886C>T (p.Gln296)	94	92	89	66	60	0
Neural	2	TMPRSS3	c.413C>A (p.Ala138Glu)	98	100	-	-	88	1
		TMPRSS3	c1276G>A (p.ala426Thr)						
	3	TMPRSS3	c.413C>A (p.Ala138Glu)	72	30	92	12	60	-1
	4	TMPRSS3	c.413C>A (p.Ala138Glu)	83	61	90	92	58	0

**Table 5:** Post-operative z-score of patients with identified variance of unknown significance.

		VUS Mutations		
Partition	Patient #	Gene	Mutation	Combined Z Score
Sensory	5	CEACAM16	c.870G>A (p.Thr290Thr)	1
		ACTG1	c.445A>G (p.Thr149Ala)	
	6	MYH9	c.5575G>A (p.Glu1859Lys)	0
	7	MYO6	c.1045A>T (p.Asn349Tyr)	0
	8	ACTG1	c.424C>G (p.Leu142Val)	1
	9	TPRN	c.2116G>a (p.Glu706Lys)	0
Neural	10	EDN3	c.43T>G (p.Ser15Ala)	1
	11	TMC1	c.1627G>A (p.Asp543Asn)	0
	12	WFS1**	c.400G>A (p.Ala134Thr)	0
		WFS1**	c.728C>T (p.Ala243Val)	
		WFS1**	c.1633G>A (p.Val545Met)	
Neural and Sensory	13	MYO6	c.2836C>T (p.Arg946Cys)	0
		OTOGL	c.3704G>A (p.Arg1235Lys)	
		OTOGL	c.5033A>G (p.ASN1678Ser)	
		DIAPH3*	c.1763C>T (p.Pro588Leu)	
		DIAPH3*	c.701T>C (p.Ile234Thr)	

* In this study we have not established if the patient carried the variants on both alleles (in trans) or in one allele (in cis).

** In this study we have not established if the patient carries this variant on both alleles (in trans) or if the variant appears to be homozygous due to its presence on only one allele with failure to detect the other parental allele for technical or genetic reasons (for example due to a SNP interfering with the assay)

## Discussion

After analyzing the genetic profile of 36 patients, our results give insight into the clinical impact of a patient’s genetic profile on cochlear implant performance. Shearer, et al. 2017 demonstrated inferior post-operative cochlear implant outcomes in patients with mutations in the neural partition after comparing outcomes of patients with mutations in the sensory partition to those with mutations in the neural partition genes OPA1, DIAPH3, DFNB59, AIFM1, TBC1D24, MTRNR1, and TMPRSS3 [6]. In our study, with only 1 neural partition gene (TMPRSS3) demonstrating a pathogenic mutation out of the panel of 28 studied, and only 1 sensory partition gene (POU4F3) demonstrating a pathogenic mutation out of the panel of 26 studied, the low sampling of genes of interest do not allow inferences to be made around the performance of patients with neural vs. sensory partition mutations as a whole. Our patient results do however build upon previously demonstrated findings regarding mutation prevalence and offer new questions into the post-operative outcomes of TMPRSS3 (Table 4). Despite being classified as a neural partition gene, patients with this mutation in our study performed quite well after cochlear implantation, even in a patient who demonstrated two separate TMPRSS3 mutations. This is in keeping with recent literature evaluating the association of genetic diagnosis for childhood-onset hearing loss on cochlear implant outcomes, in which patients with a TMPRSS3 mutation demonstrated some of the highest speech perception outcomes [13].

Our inclusion criteria into neural or sensory partition groups employed more stringent criteria than previously published studies that permitted categorization of patients with genetic variants based on CADD score analysis alone, rather than categorizing patients based on pathogenicity utilizing ACMG guidelines [6]. Utilizing an aggregate score from several in- silico programs, the CADD score analysis is an appropriate method for whole exome or genome exploration to rapidly screen genes that may fit the phenotype of interest before a detailed analysis. CADD score analysis alone is not able to classify a variant as pathogenic however, making more stringent categorization criteria appropriate when building upon genetic screening studies. Our utilization of the ACMG guidelines in the classification of the variants increases the rigor of the results, although it also decreases the number of patients available for statistical analysis. Our incidence of pathogenic mutations was 11%, and incidence of isolated or combined neural/sensory VUS was 25%. The low rate of identified pathogenic mutations demonstrates the challenges with attaining adequately powered genetic studies at present, and need for further VUS study. As more pathogenic mutations are identified and classified from VUS, it will increase the power of future studies. The mutations of the 25% of patients with VUS have been listed to facilitate future characterization of pathogenicity if additional functional evidence becomes available (Table 5).

Moving towards genetic profiles as a means of predicting cochlear implant performance, both high risk genes, and high-risk variants within those genes, must be understood to determine potential post-operative outcomes. The TMPRSS3 gene harbored all of the neural partition pathogenic mutations in our study, with a higher incidence also seen in prior literature [6]. Encoding a type III transmembrane serine protease, the TMPRSS3 gene has had 87 different variants described associated with non-syndromic hearing loss, with native gene expression seen in inner hair cells, spiral ganglion neurons, stria vascularis, and the cochlear aqueduct [14, 15]. Within our study, the three patients with pathogenic TMPRSS3 gene mutations showed variable post-operative performance. One patient was a poor performer with a z-score of -1, one patient performed average with a z-score of 0, and interestingly, the last patient demonstrated 2 separate pathogenic mutations in the TMPRSS3 gene yet demonstrated relatively good performance with a Z-score of 1. Previous research into post-operative outcomes of patients with TMPRSS3 mutations has been mixed, however one of the most recent and largest reviews of the topic by Seok Moon et al did show excellent outcomes following cochlear implantation [14, 16]. In addition to other plausible explanation for these differences, such as duration of deafness and post-operative rehabilitation engagement, there may also be specific variants within the same gene that display a unique post-operative phenotype, especially with the gene demonstrating expression in multiple areas of the inner ear. This could be driven by variable functional consequences in-vivo at different anatomical locations. For example, the patient who demonstrated good performance was compound heterozygote for two variants located in different domains of the TMPRSS3 protein. The c.1276G>A variant has been shown to be associated with varying degrees of reduced activity in a functional assay measuring proteolytic activity. The second variant c.413C>A (p.Ala138Glu) is present in the SPCR domain of the protein and is associated with protein-protein interactions. Although both variants are pathogenic, the clinical impact each mutation imposes on cochlear implant performance may be variable. In addition, the different outcomes in patients with homozygous TMPRSS3 c.413C>A variants may be due to other, not yet well understood, co-existing variants. For example, the worse performing patient with TMPRSS3 c.413C>A variant was also heterozygous for the MYO6 c.2449T>G (p.Cys817Gly), classified as VUS, which in certain families has been shown to be inherited in a autosomal dominant mode. It is possible that their poor performance could therefore be due to an increased burden of genetic variants in other genes.

Factors that contribute to postoperative speech perception outcomes in adults with post-lingual deafness include the amount of residual hearing, duration of deafness, neurocognitive functioning, type of device implanted, method of implantation, surgeon experience, postoperative complications, and environmental variables such as socioeconomic status [17, 18]. Over the last two decades, mean post-operative performance levels on tests of speech perception in quiet and noise have improved steadily as technology improves, however poor performers still exist [19]. Genetic factors are suggested to be the single largest independent predictor of post-operative outcomes [6, 20]. With technological progression occurring rapidly, and cochlear implantation being provided to patients with residual hearing, there is a demand for extensive genetic testing in patients not just for cochlear implant candidacy, but also as a mechanism to understand natural hearing loss progression, the expected degree of natural hearing preservation, and outcomes to various auditory rehabilitation strategies [21]. As demonstrated in our study, sensory and neural partition pathogenic variants may help provide useful post-operative information for patients, however the molecular mechanisms underlying the genes and variants involved in non-syndromic hearing loss add additional complexity when understanding post-operative implications. Future high powered genetic cochlear implant outcome studies will help drive this field forward [22]. Patients with genetic mutations that lack benefit from a cochlear implant would warrant high priority for future gene therapy and auditory brainstem implantation consideration [23, 24].

## Conclusions

While patients with pathogenic variants in neural partition genes have been shown to have worse CI performance, not all pathogenic mutations within these gene are equal when evaluating cochlear implant outcomes. Patients with pathogenic TMPRSS3 mutations may demonstrate favourable CI results despite neural partition expression.

## Supporting information

S1 File Dataset. (PDF)
