# Supplementary material for: Gene mutations as a non-invasive measure of adult cochlear implant performance: Variable outcomes in patients with select TMPRSS3 mutations
Source: PLoS One. 2023 Sep 15;18(9):e0291600. doi: 10.1371/journal.pone.0291600 (PMC10503761; doi:10.1371/journal.pone.0291600)
Supplement: S1 File — (PDF) [file pone.0291600.s001.pdf]

| [redacted] | HINQ | HINTN | AZQ | AZN | CNC | Avg Zscore | GenesPathogenicControl (sensory) | GenesPathogenicTest (Neural) | GenesVUSControl | GenesVUSTest |
|---|---|---|---|---|---|---|---|---|---|---|
| [redacted] | 53 | 47 | | | 26 | -2 | 0 | 0 | 0 | 0 |
| [redacted] | 93 | | 100 | 97 | 84 | 1 | 0 | 0 | 1 | 0 |
| [redacted] | 98 | 61 | 93 | 50 | 64 | 0 | 0 | 0 | 1 | 1 |
| [redacted] | 77 | 33 | 41 | | 40 | -2 | 0 | 0 | 0 | 0 |
| [redacted] | 98 | 96 | 97 | 90 | 88 | 1 | 0 | 0 | 0 | 0 |
| [redacted] | | | 99 | 86 | 78 | 1 | 0 | 0 | 0 | 0 |
| [redacted] | 96 | 79 | 92 | 73 | 74 | 0 | 0 | 0 | 1 | 0 |
| [redacted] | | | 98 | 100 | 88 | 1 | 0 | 1 | 0 | 0 |
| [redacted] | 91 | 92 | 92 | 66 | 78 | 1 | 0 | 0 | 0 | 1 |
| [redacted] | 92 | 12 | 72 | 30 | 60 | -1 | 0 | 1 | 0 | 0 |
| [redacted] | 98 | 77 | 70 | 41 | 42 | 0 | 0 | 0 | 0 | 1 |
| [redacted] | 87 | 70 | 85 | 79 | 72 | 0 | 0 | 0 | 0 | 1 |
| [redacted] | | | 95 | 71 | 66 | 0 | 0 | 0 | 0 | 0 |
| [redacted] | 90 | 92 | 83 | 61 | 58 | 0 | 0 | 1 | 1 | 0 |
| [redacted] | 98 | 94 | 88 | 57 | 68 | 0 | 0 | 0 | 0 | 0 |
| [redacted] | 89 | 66 | 94 | 92 | 60 | 0 | 1 | 0 | 0 | 0 |
| [redacted] | 50 | | | | 16 | -3 | 0 | 0 | 0 | 0 |
| [redacted] | | | 95 | 75 | 84 | 1 | 0 | 0 | 0 | 0 |
| [redacted] | 89 | 44 | 38 | 12 | 32 | -1 | 0 | 0 | 0 | 0 |
| [redacted] | 96 | 52 | 100 | 23 | 68 | 0 | 0 | 0 | 0 | 0 |
| [redacted] | 98 | 61 | 80 | 9 | 44 | -1 | 0 | 0 | 0 | 0 |
| [redacted] | | | 97 | 79 | 78 | 1 | 0 | 0 | 0 | 0 |
| [redacted] | 96 | 77 | 92 | 50 | 52 | 0 | 0 | 0 | 0 | 0 |
| [redacted] | 100 | 62 | 92 | 44 | 60 | 0 | 0 | 0 | 1 | 0 |
| [redacted] | 86 | 74 | 51 | 12 | 56 | -1 | 0 | 0 | 0 | 0 |
| [redacted] | 100 | 61 | 60 | 0 | 38 | -1 | 0 | 0 | 0 | 0 |
| [redacted] | 96 | 87 | 84 | 79 | 68 | 0 | 0 | 0 | 0 | 0 |
| [redacted] | 96 | 88 | 93 | 72 | 88 | 1 | 0 | 0 | 0 | 0 |
| [redacted] | | | 87 | 46 | 40 | 0 | 0 | 0 | 0 | 0 |
| [redacted] | | | 98 | 75 | 32 | 0 | 0 | 0 | 0 | 0 |
| [redacted] | 100 | 100 | 100 | 83 | 84 | 1 | 0 | 0 | 1 | 0 |
| [redacted] | | | 86 | 34 | 48 | 0 | 0 | 0 | 0 | 0 |
| [redacted] | 98 | 82 | | | 80 | 1 | 0 | 0 | 0 | 0 |
| [redacted] | 100 | 76 | 97 | 34 | 60 | 0 | 0 | 0 | 0 | 0 |
| [redacted] | | | 71 | | 60 | 0 | 0 | 0 | 0 | 0 |
| [redacted] | 98 | 84 | 92 | 46 | 50 | 0 | 0 | 0 | 1 | 0 |
| **Average Score** | **91** | **71** | **85** | **57** | **61** | | | | | |
| **Std** | **12.66** | **21.29** | **16.63** | **27.97** | **19.18** | | | | | |
| **n** | **27** | **25** | **33** | **31** | **36** | | | | | |
